# Weighted Genetic Risk Scores and Prediction of Weight Gain in Solid Organ Transplant Populations

**DOI:** 10.1371/journal.pone.0164443

**Published:** 2016-10-27

**Authors:** Núria Saigi-Morgui, Lina Quteineh, Pierre-Yves Bochud, Severine Crettol, Zoltán Kutalik, Agnieszka Wojtowicz, Stéphanie Bibert, Sonja Beckmann, Nicolas J Mueller, Isabelle Binet, Christian van Delden, Jürg Steiger, Paul Mohacsi, Guido Stirnimann, Paola M. Soccal, Manuel Pascual, Chin B Eap, the Swiss Transplant Cohort Study

**Affiliations:** 1 Unit of Pharmacogenetics and Clinical Psychopharmacology, Department of Psychiatry, Lausanne University Hospital, Prilly, Switzerland; 2 Service of Infectious Diseases, University Hospital and University of Lausanne, Lausanne, Switzerland; 3 Institute of Social and Preventive Medicine (IUMSP), Lausanne University Hospital, Lausanne, Switzerland; 4 Swiss Institute of Bioinformatics, Lausanne, Switzerland; 5 Institute of Nursing Science, University of Basel, Basel, Switzerland; 6 Division of Infectious Diseases and Hospital Epidemiology, University Hospital, Zurich, Switzerland; 7 Service of Nephrology and Transplantation Medicine, Kantonsspital, St Gallen, Switzerland; 8 Service of Infectious Diseases, University Hospitals, Geneva, Switzerland; 9 Service of Nephrology, University Hospital, Basel, Switzerland; 10 Swiss Cardiovascular Center Bern, University Hospital, Bern, Switzerland; 11 University Clinic of Visceral Surgery and Medicine, Inselspital, Bern, Switzerland; 12 Service of Pulmonary Medicine, University Hospital, Geneva, Switzerland; 13 Transplant Center, Lausanne University Hospital, Lausanne, Switzerland; 14 School of Pharmaceutical Sciences, University of Geneva, University of Lausanne, Geneva, Switzerland; University Hospital Jena, GERMANY

## Abstract

**Background:**

Polygenic obesity in Solid Organ Transplant (SOT) populations is considered a risk factor for the development of metabolic abnormalities and graft survival. Few studies to date have studied the genetics of weight gain in SOT recipients. We aimed to determine whether weighted genetic risk scores (w-GRS) integrating genetic polymorphisms from GWAS studies (SNP group#1 and SNP group#2) and from Candidate Gene studies (SNP group#3) influence BMI in SOT populations and if they predict *≥*10% weight gain (WG) one year after transplantation. To do so, two samples (n_A_ = 995, n_B_ = 156) were obtained from naturalistic studies and three w-GRS were constructed and tested for association with BMI over time. Prediction of 10% WG at one year after transplantation was assessed with models containing genetic and clinical factors.

**Results:**

w-GRS were associated with BMI in sample A and B combined (BMI increased by 0.14 and 0.11 units per additional risk allele in SNP group#1 and #2, respectively, p-values<0.008). w-GRS of SNP group#3 showed an effect of 0.01 kg/m^2^ per additional risk allele when combining sample A and B (p-value 0.04). Models with genetic factors performed better than models without in predicting 10% WG at one year after transplantation.

**Conclusions:**

This is the first study in SOT evaluating extensively the association of w-GRS with BMI and the influence of clinical and genetic factors on 10% of WG one year after transplantation, showing the importance of integrating genetic factors in the final model. Genetics of obesity among SOT recipients remains an important issue and can contribute to treatment personalization and prediction of WG after transplantation.

## Introduction

Obesity has become a worldwide major concern since it has more than doubled in the last decades. In 2014, 39% of adults were overweight (25 kg/m^2^≤ Body Mass Index (BMI)< 30 kg/m^2^) and 13% were obese (BMI ≥ 30 kg/m^2^) [1]. Obesity is a risk factor leading to other co-morbidities such as diabetes, cardiovascular diseases and certain type of cancers [1]. Among solid organ transplant (SOT) recipients, the rate of overweight and obesity has increased over the past years. By 2011, 34% of liver transplant candidates were obese, compared to 29% in 2001 [2]. Similar results have been found for kidney, heart and lung transplant recipients [3–5]. Although overweight and obesity prevalence are similar to those measured in general population studies, in SOT recipients the consequences are more serious. Indeed, obesity in SOT is an important risk factor for the development of New Onset Diabetes after Transplant (NODAT) [6] which has a deleterious effect on graft survival [7, 8]. Moreover, it can be often associated with delayed graft function related to surgical and post‐operative complications [9].

Few longitudinal studies examining weight gain (WG) among transplant recipients have been conducted to date, most of them focusing on WG during the first year post transplantation. A WG ranging from 3.5 to 10 Kg has been reported in heart, liver and kidney transplant recipients [10–14] and a mean of 10% WG during the first year after transplantation was described in kidney transplant recipients [15]. A threshold of 10% increase of ideal body weight, defined as the metropolitan relative weight criteria [16], has been related to a risk of developing cardiovascular disease in general populations followed for more than 25 years [16, 17]. Ethnicity, sex, age in addition to specific factors such as transplanted organ, glucocorticoids and immunosuppressive treatments are some of the described factors influencing WG following SOT and NODAT [18], as well as genetic factors. Most studies on transplant populations focused mainly on the NODAT rather than WG [19, 20]. Regarding BMI-related phenotypes, a protocol for the first systematic literature review has been published. The aim is to condense and compare the current state of evidence on WG, overweight and obesity in SOT individuals including genetic and non-genetic factors [21]. Regarding candidate gene approach, two single nucleotide polymorphisms (SNPs) and one insertion/deletion have previously been associated with BMI-related phenotypes [22–24]. These studies were conducted in three heterogeneous populations with small or moderate sample sizes (n<270), with different obesity-related outcomes and type of transplanted organ. Furthermore, different polymorphisms were analyzed. To our knowledge, no Genome Wide Association Studies (GWAS) investigating BMI variants within SOT recipients have yet been published. Recently, a microarray study examining gene expression in subcutaneous adipose tissue in kidney transplant recipients found that the expression of obesity-related genes was correlated with weight change [25]. The top 41 ranked genes were further associated with obesity through a text mining approach [26], including genes related to diabetes, obesity and neurological concepts such as dopamine, nicotine, and cognition [25]. Interestingly, two of these genes (i.e. *MTCH2* and *TFAP2B*) were also found in the largest BMI GWAS meta-analysis conducted to date in the general population [27]. This meta-analysis was conducted in more than 300 000 individuals and reported 97 SNPs associated with BMI, also including 32 previously replicated BMI SNPs [28–31]. All 97 polymorphisms explained up to 2.7% of BMI variability within these individuals [27]. Since polygenic or common obesity is influenced by many genetic polymorphisms, genetic risk scores (GRS) provide a useful tool summarizing risk-associated variations across the genome by aggregating information from multiple-risk SNPs, and they may improve the consistency and the power to determine genetic risk in polygenic diseases [32, 33].

In the present study, we aimed to study the association of three weighted GRS, integrating previously published SNPs, with BMI in two cohorts of Swiss transplanted individuals. In addition, we assessed whether these genetic polymorphisms could predict a *≥*10% WG during the first year post transplant.

## Materials and Methods

### Sample A

The Swiss transplant cohort study (STCS) is an ongoing prospective multicenter study (Basel, Bern, Geneva, Lausanne, St. Gallen and Zurich) started in May 2008 which enrolls SOT recipients with no particular eligibility or exclusion criteria other than having received an allotransplant and having signed the informed consent [34]. The present study (May 2008–May 2011) included SOT recipients (i.e. kidney, liver, lung, heart, or multi-organ) with a functional graft for at least 12 months after transplantation in order to have a sufficient period of follow-up (A total of 1294 patients were followed up in their respective transplant centers at baseline and at 6, 12, 24, 36 and 48 months after transplantation. Lipid profile, BMI, blood pressure and patient characteristics were collected at the different time-points of the follow up. Further details have been published elsewhere [34, 35]. Only Caucasians and Recipients of 18 years or older were retained. If an individual was subjected to more than one transplant, only the first SOT was considered. A total of 995 patients were considered for analysis.

### Sample B

A total of 197 SOT recipients (i.e. lung, liver and kidney) were enrolled between 2003 and 2005 from the outpatient clinic of the transplant center of the University Hospital of Lausanne, Switzerland. Only patients with a functional graft for more than 12 months were eligible to participate in the study. Further details can be found elsewhere [35–37]. Briefly, data regarding patients’ age, gender, BMI, ethnicity, immunosuppressive treatments among others were collected retrospectively from the medical files. Additionally, data concerning weight, at baseline, at 1, 3, 6, 9, 12 and at the yearly follow-up during the 5 years after transplantation were collected retrospectively from the medical files between October 2011 and April 2012. Blood samples were collected for further genotyping analysis. 156 individuals of 18 years or older for whom Caucasian ethnicity was reported and had clinical data available, were included in the analysis. This sample was considered as a replication sample.

All patients gave their written informed consent and the studies were approved by the ethics committee of the Lausanne and Geneva University Hospitals.

### Genotype selection and genotyping

SNP selection was done according to large Meta analyses of GWAS published on BMI. SNP group#1 included 32 BMI associated polymorphisms in general adult populations [28]. A second group consisted of 97 SNPs (SNP group#2) recently associated with BMI in general populations and which included the previous 32 SNPs (or its proxies) [27]. Only SNPs significant at GWAS levels (i.e. p-value < 5x10^-8^) were retained for the analysis. S1 and S2 Tables show a detailed description of the selected SNPs.

Additionally, 41 genes whose expression in subcutaneous adipose tissue has been previously associated with weight change in kidney transplant recipients [25] were included. A selection of tagging SNPs of these genes was obtained using HapMap Genome Browser (release 28). In order to avoid over representation of a particular gene, one tagging SNP per gene was selected based on the number of SNPs tagged and on the genotype availability in our samples. Six genes were excluded since no tagging SNPs were found in HapMap. Of note, two genes (i.e. *MTCH2*, *TFAP2B*) were also present in the GWAS mentioned previously (SNP group#1). Finally 19 SNPs, for which genotype was available in both samples A and B, were retained in the SNP group#3. A detailed description of these genes and polymorphisms can be found in S3 Table.

For the sample A, genotypes were analyzed with the Human OmniExpress-24 BeadChip Kit as described by the manufacturer’s protocol (Illumina, San Diego, CA). For the sample B, genotyping was performed using the Illumina 200K Cardiometabochip (Illumina, San Diego, CA). Briefly, the CardioMetabochip is a custom Illumina iSelect genotyping array designed to test DNA variation of 200’000 SNPs from regions identified by large scale meta-analyses of genome wide association studies (GWAS) for metabolic and cardiovascular traits [38]. Polymorphisms or proxies were chosen based on genotype availability. A Quality Control was done for the genotyped SNPs. Samples were excluded from the analysis if sex was inconsistent with genetic data from X-linked markers, and when genotype call rate was <0.96 and gene call score <0.15. GenomeStudio Data Analysis Software was used to export results generated by Illumina CardiometaboChip.

### Construction of Genetic Risk Scores

Three GRS were built following a weighted GRS (w-GRS) method as previously described [28] with 32 SNPs (SNP group#1) and 97 SNPs (SNP group#2) both from GWAS, and 19 SNPs (SNP group#3) from candidate genes. Briefly, genotypes from each SNP were coded as 0, 1 or 2 according to the number of BMI risk alleles and each polymorphism was then weighted by its β-coefficient (allele effect) based on the assumption that all SNP of interest have independent effects and contribute in an additive manner on BMI. In order to facilitate interpretation, the GRS was subsequently rescaled as previously described [39]. Thus, each unit increase in the GRS corresponded approximately to one additional risk allele. Allele effects on BMI were obtained from those published in the literature for the SNPs group#1 and #2 [27, 28]. For the SNP group#3 allele effects were calculated from a large population based sample, GIANT, which consisted in a meta-analysis of GWAS with a discovery set of 123,865 individuals of European ancestry from 46 studies for height [40], BMI [28] and waist-to-hip ratio [41].

### Statistical analysis

Descriptive analysis of quantitative data is presented as median and range unless otherwise specified whereas qualitative data is expressed as percentages. Chi-squared test or rank sum test were used for association studies within categorical data or non-parametric continuous variables, respectively. Hardy-Weinberg Equilibrium (HWE) was determined for each polymorphism by a chi-square test. P-value threshold was set at <0.05 and Bonferroni multiple test correction was applied when necessary (i.e. 0.05/6; 2 tests of w-GRS in the whole sample A and 2 tests of w-GRS in two different subgroups). Due to the exploratory nature of the analysis conducted for the third GRS, in the present work we did not consider multiple test correction for this GRS.

For multivariate analysis, a Generalized Additive Mixed Model (GAMM) was used to deal with complex and non-linear BMI evolution at different time points and presence of multiple observations per individual introducing interdependence among observations. A random effect at the subject level was also introduced to take the dependence structure of observed data into account. The GAMMs were fitted using the mgcv package of R (settings were fixed at package defaults). To be more conservative, the uncertainty of estimated parameters was assessed by 1’000 bootstraps on individuals [42]. Multivariate models were adjusted by gender, type of treatment, organ, living donor and CMV as previously described in the literature [18], as well as genetic factors and time of follow-up. Because sex and age have been described as factors influencing WG [15], further analyses were conducted stratifying by gender and the median age when the interactions with w-GRS were significant.

### Prediction of ≥10% WG one year after transplantation in the sample A

A binary logistic regression model at 12 months after transplant was used to determine whether clinical and genetic factors influence a *≥*10% WG one year after transplantation for those cases where genetic components were significantly associated with BMI. The ability to discriminate between gainers of 10% weight versus those who did not gain 10% one year after transplantation was assessed with the Area Under the Receiver Operating Characteristic Curve (AUROC) for a model containing only clinical covariates (i.e. age, sex, transplanted organ, BMI at baseline, immunosuppressant treatment) and another model integrating clinical and genetic factors. For each pair of models compared (i.e. the non-genetic nested in its corresponding genetic model) the same number of individuals must be tested in order to be comparable. In addition, Sensitivity (percentage of correctly predicted individuals with ≥10% WG among all individuals with ≥10% WG), Specificity (percentage of correctly predicted individuals with <10% WG among all truly individuals with <10% WG) and Accuracy (percentage of correctly classified gainers of ≥10% weight among all subjects) were obtained for each model using “pROC” R package [43]. An AUROC lower than 0.70 indicates low discriminative accuracy [44]. As previously described, [45, 46] in order to assess the added value of selected SNPs in predicting a *≥*10% WG one year after transplantation (i.e. comparison of genetic and non-genetic models), likelihood ratio tests (LRT) and Integrated Discrimination Improvement (IDI) estimates with their respective p-values were calculated. Finally, the number needed to genotype (NNG) (i.e. the average number of patients who need to be genotyped to detect one misclassified case of ≥10% WG one year after transplantation if using only clinical covariates) was calculated based on the inverse of the difference between the accuracy of clinical and genetic models [47].

## Results

### Population description

**Fig 1:**
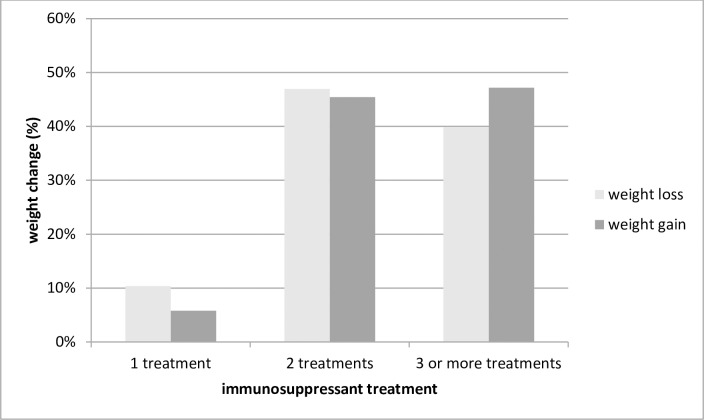
Percentage of weight gain in Sample A at one year after transplantation by number of immunosuppressant treatments (cyclosporine, tacrolimus, glucocorticoids, azatioprine and/or mycophenolate).

The characteristics of sample A are presented in Table 1. Sixty-six percent were men, 17.0% were obese one year after transplantation and 27.1% were diagnosed of NODAT. Similar patterns (p>0.05) were observed in sample B (60.9%, 18.5% and 28.8%, respectively, Table 2). Twenty three percent of individuals in sample A gained ≥10% of weight the first year after transplantation and 35% of individuals in sample B (p<0.001). The mean of WG one year after transplantation was 3.5% and 6.3% for samples A and B, respectively. Sample A included also heart and multi-organ transplant, individuals were older than in sample B (median age: 54 years compared to 48, p<0.001) and there was a high prevalence of living donors (27.1% and 11.5%, respectively, p<0.001). Tacrolimus (TAC) was more frequently prescribed in sample A, whereas cyclosporine (CSA) was more used in sample B (45.1% versus 34.6%, respectively for TAC and 19.6% versus 65.4%, respectively for CSA; p<0.05). For sample A, individuals with at least 3 immunosuppressive treatments (i.e. cyclosporine, tacrolimus, glucocorticoids, azatioprine and/or mycophenolate) gained significantly more weight at one year after transplantation compared to the others (p = 0.01, Fig 1). Of note, 99% of those with at least 3 immunosuppressants had a glucocorticoid treatment prescribed, possibly contributing to this weight gain. No significant results were found in sample B. Among those individuals with less than 3 immunosuppressant drugs, sample A had lower prescription of glucocorticoids than sample B, probably contributing to explain the differences of weight gain observed between both samples (S4 and S5 Tables).

**Table 1:** Characteristics of Sample A (all and by 10% weight gain one year after transplantation).

Characteristic	All	wg≥10%*	wg<10%*	p-value #
	n = 995	n = 204	n = 673	
Recipient age at transplantation (years), median (range)	54 (18–79)	51 (18–73)	55 (18–79)	**0.0001**
Recipient men (%)	66.0	56.8	68.9	**0.001**
Period of follow up (months), median (range)	12 (0–48)	12 (0–48)	12 (0–48)	0.55
Living donor (%)	27.1	27.9	29.6	0.6
Donor age (years), median (range)	53 (1–86)	50 (1–80)	53 (1–86)	**0.04**
Transplanted organ (%)				
Kidney	62.4	61.3	67.2	**<0.001**
Liver	15.9	10.3	14.7	
Lung	9.5	14.7	7.8	
Heart	6.5	11.3	4.6	
Multi-organ transplantation	4.1	2.5	4.5	
**Before transplant**				
BMI (kg/m^2^), median (range)	24.6 (13.7–41.2)	23.1 (14.9–37.4)	24.9 (14.3–41.2)	**0.0001**
Overweight (25 kg/m^2^ ≤ BMI >30 kg/m^2^), %	30.7	23.0	32.5	**<0.001**
Obese (BMI ≥ 30 kg/m^2^), %	15.3	9.3	16.8	
HDL (mmol/L), median (range)	1.2 (0.01–8)	1.2 (0.1–4.1)	1.2 (0.09–8)	0.7
LDL (mmol/L), median (range)	2.2 (0.06–10.02)	2.2 (0.1–7.1)	2.2 (0.08–10.0)	0.3
Cholesterol (mmol/L), median (range)	4.2 (0.3–11.7)	4.0 (0.3–9.9)	4.2 (0.8–11.7)	0.2
**At 12 months after transplant**				
BMI (kg/m^2^), median (range)	25.2 (15.3–44.6)	27.1 (18.8–44.6)	24.7 (15.3–44.3)	**0.0001**
Overweight (25 kg/m^2^ ≤ BMI >30 kg/m^2^), %	34.7	39.0	33.0	**<0.001**
Obese (BMI ≥ 30 kg/m^2^), %	17.0	27.0	14.0	
HDL (mmol/L), median (range)	3.5	1.3 (0.5–4.1)	1.3 (0.2–7.0)	0.08
LDL (mmol/L), median (range)	1.3 (0.21–7)	2.6 (0.8–5.8)	2.6 (0.3–8.7)	0.8
Cholesterol (mmol/L), median (range)	2.6 (0.3–8.7)	5.0 (2.3–9.2)	4.8 (1.7–12.0)	**0.01**
Incidence of NODAT (%)$	27.1	25.9	28.1	0.6
**CMV serostatus (%)**				
Recipient CMV infection (R+)	57.1	21.8	23.7	0.9
Donor CMV infection (D+)	53.0	20.8	20.6	
Recipient and Donor CMV infection (R+D+)	32.6	33.2	33.1	
**Calcineurin inhibitors (%)**				
TAC	45.1	42.2	48.6	0.3
CSA	19.6	21.1	19.6	
None	35.2	36.8	31.8	

wg: weight gain, CMV: Cytomegalovirus, TAC: Tacrolimus, CSA: Cyclosporine, NODAT: New Onset Diabetes After Transplant

# comparison between wg≥10% and wg<10%

*at 12 months after transplantation, missing n = 118

$NODAT was diagnosed if patients were taking an antidiabetic treatment after transplantation or if diabetes was reported in their case report forms. NODAT excluded those patients with diabetes previous to transplant

**Table 2:** Characteristics of Sample B (all and by 10% weight gain one year after transplantation).

Characteristic	All	wg≥10%*	wg<10%*	p-value #
	156	42	78	
Recipient age at transplantation (years), median (range)	48 (22–68)	47 (26–66)	49 (22–68)	0.4
Recipient men (%)	60.9	59.5	61.5	0.8
Period of follow up (months), median (range)	12 (1–60)	12 (1–60)	12 (1–60)	1
Living donor (%)	11.5	11.9	7.7	0.4
Donor age (years), median (range)	43.5 (10–73)	45 (10–65)	43 (11–69)	0.7
Transplanted organ (%)				
kidney	65.4	76.2	60.3	**0.03**
Liver	23.7	7.1	26.9	
Lung	10.9	16.7	12.8	
**Before transplant**				
BMI (kg/m2), median (range)	23.4 (15.8–37.3)	22.9 (18.7–33.5)	24.2 (15.8–37.3)	0.06
Overweight (25 kg/m^2^ ≤ BMI >30 kg/m^2^), %	24.1	14.3	30.8	0.08
Obese (BMI ≥ 30 kg/m^2^), %	10.9	9.5	12.8	
**At 12 months after transplant**				
BMI (kg/m2), median (range)	25.2 (16.5–39.3)	26.8 (20.9–39.3)	24.3 (16.5–35.4)	**0.0006**
Overweight (25 kg/m^2^ ≤ BMI >30 kg/m^2^), %	35.1	45.2	28.2	**0.004**
Obese (BMI ≥ 30 kg/m^2^), %	18.5	28.6	14.1	
Incidence of NODAT (%)	28.8	30.9	35.9	0.6
**CMV serostatus (%)**				
Recipient CMV infection (R+)	49.3	30.8	36.1	0.6
Donor CMV infection (D+)	61.5	23.1	15.3	
Recipient and Donor CMV infection (R+D+)	27.6	30.8	27.8	
**Calcineurin inhibitors (%)**				
TAC	34.6	26.2	47.4	**0.02**
CSA	65.4	73.8	52.7	

wg: weight gain, CMV: Cytomegalovirus, TAC: Tacrolimus, CSA: Cyclosporine, NODAT: New Onset Diabetes After Transplant.

# comparison between wg≥10% and wg<10%

*at 12 months after transplantation, missing n = 36

#### 10% WG one year after transplantation

In both samples A and B, those gaining ≥10% of weight had lower BMI at baseline and higher BMI 12 months after transplantation compared to those gaining <10% (Tables 1 and 2). The prevalence of overweight and obese was lower at baseline and higher at one year after transplantation for ≥10% when compared to <10% WG. The transplanted organ differed between ≥10% and <10% for both A and B samples. The kidney was the most prevalent transplanted organ in both groups. The second most prevalent transplanted organ in the ≥10% WG group was the lung while the heart and the liver were the third most frequently transplanted organs. In the <10% WG group, the liver and the lung were among the second and the third most frequently transplanted organs. Additionally, in sample A donors were younger and individuals had higher cholesterol levels at 12 months in the ≥10% WG group (median: 50 years and 5.0 cholesterol mmol/L, p 0.04 and p 0.01, respectively). In sample B, significant differences were found in the prescribed immunosuppressive treatments; CSA was highly prescribed in the ≥10% WG when compared to the <10% WG group (73.8% versus 52.7%, respectively; p 0.02).

### Genetic Risk Score analysis

#### Weighted genetic risk score with GWAS polymorphisms

In samples A and B, w-GRS ranged from 16 to 40 (SNP group#1) and from 63 to 107 (SNP group#2), respectively. S1 Fig shows the w-GRS distribution percentage in each sample. The association between w-GRS and BMI over time for sample A is shown on Table 3. w-GRS built from the SNP group#1 was significantly associated with BMI, showing a 0.16 BMI units increase per additional risk allele and an explained variability of 1.46%. When stratified by the median of age (w-GRS*age p = 0.001 and p = 0.02 for SNP group#1 and #2, respectively) individuals older than 54 years old had 0.23 BMI unit increase per additional risk allele and an explained BMI variability of 2.74% whereas those at 54 years or younger showed a trend of 0.10 units increase and 0.56% of explained BMI variability after multiple test correction (p = 0.08). For SNP group#2, the effect was slightly lower (0.11 units of BMI per risk allele increase, explained variability of 2.08%). These results could be partially replicated in sample B (Table 4) for SNP group#1 with an effect of 0.20 BMI units per risk allele increase and explained variability of 2.40%. Analysis stratified by sex (w-GRS*sex, p = 0.03 for SNP group#1) showed no significant associations after multiple test correction (Table 4). Additionally, a significant interaction between w-GRS and organ (i.e. kidney/non kidney) was found for sample B and SNP group#1 (n = 83, p-value 0.04) showing a slightly higher effect (0.30 units of BMI per risk allele increase) in kidney transplanted individuals when compared to the overall 0.20 units. When combining samples A and B, BMI increased by 0.14 [0.09–0.19] and 0.11 [0.07–0.15] units per additional risk allele in SNP group#1 and #2, respectively, p-values<0.001).

**Table 3:** Weighted Genetic Risk Scores from GWAS SNPs and their associations with BMI in Sample A.

	n	Effect on BMI per additional risk allele [CI 95%]	p-value*	E. Var (%)
**SNP group#1**				
All population	881	0.16 [0.11–0.23]	**p<0.008**	1.46
Age [18–54] years	444	0.10 [0.01–0.17]	*0*.*08*	0.56
Age > 54 years	437	0.23 [0.14–0.32]	**p<0.008**	2.74
**SNP group#2**				
All population	854	0.11 [0.08–0.15]	**p<0.008**	2.08
Age [18–54] years	452	0.08 [0.03–0.13]	**p<0.008**	1.10
Age > 54 years	426	0.13 [0.07–0.19]	**p<0.008**	2.90

E. Var: Explained Variability

CI: Confidence Interval

SNP: Single Nucleotide Polymorphism

BMI: Body Mass Index

* p-value corrected by multiple test

**Table 4:** Weighted Genetic Risk Scores from GWAS SNPs and their associations with BMI in Sample B.

	n	Effect on BMI per additional risk allele [CI 95%]	p-value	E. Var (%)
**SNP group#1**				
All population	124	0.20 [0.07–0.35]	**0.02**	2.40
Men	82	0.14 [-0.01–0.31]	0.05*	n.c
Women	61	0.28 [-0.05–0.63]	0.05	n.c
**SNP group#2**				
All population	117	0.02 [-0.08–0.11]	0.28	n.c
Men	69	-0.03 [-0.18–0.07]	0.33	n.c
Women	53	0.04 [-0.16–0.25]	0.34	n.c

E. Var: Explained Variability

CI: Confidence Interval

BMI: Body Mass Index

SNP: Single Nucleotide Polymorphism

n.c: not calculated because of non significant association and/or low sample size

* values obtained with 100 bootstraps

#### Weighted genetic risk score in Candidate Gene polymorphisms (SNP group#3)

No association of w-GRS from SNP group#3 and BMI was found in sample A whereas an increase of 0.05 units of BMI per additional risk allele was found in sample B (p-value 0.048) with an explained BMI variability of 1.72% (S6 Table). In addition, in sample B, when SNPs group#3 and #1 were combined (49 SNPs excluding repeated SNPs) a significant association with BMI was found with an increase of 0.16 BMI units per additional risk allele and an explained BMI variability of 4.1% (p-value: 0.001) (S7 Table). The w-GRS from group SNP#3 in the combined A and B sample showed an effect of 0.01 kg/m^2^ per additional risk allele (p-value 0.04).

#### Prediction of 10% WG one year after transplantation

For the models in which the w-GRS was significantly associated with BMI, we evaluated the ability of the model to discriminate between gainers of ≥10% of weight and those who gained <10% the first year after transplantation. In sample A, a model adjusted by clinical covariates (i.e. age, sex, immunosuppressant treatment (tacrolimus and/or cyclosporine), baseline BMI and transplanted organ) as well as genetic factors (i.e. SNP group#1) performed better than a model adjusted only by clinical covariates (LRT-p: 0.0004). The predictive value for gaining 10% or more weight when including SNP group#1 in the model resulted in an AUROC of 0.74, a specificity of 0.61, a sensitivity of 0.77 and an accuracy of 0.65, whereas the model without genetic components had 0.66, 0.59, 0.66 and 0.61 of AUROC, specificity, sensitivity and accuracy, respectively (Table 5). Similarly, the genetic model including SNP group#2, performed better (LRT-p: 0.008) and had higher AUROC (0.80) than the non genetic model (AUROC non genetic: 0.66). Similarly, for sample B, the genetic model including clinical covariates and SNP group#1 was significantly different from the clinical model, (LRT-p: 0.04) had an AUROC of 0.89 and a specificity, sensitivity and accuracy of 0.78, 0.88 and 0.81, respectively (Table 5). The prediction performance of the genetic model compared to the non-genetic one was significantly improved as shown by the IDI score. A statistically significant IDI (p<0.01, sample A, SNP group#2, Table 5) means a significant improvement of the genetic model prediction, by increasing the average of sensitivity and one minus specificity of the model. The lowest NNG in order to detect one misclassified case of ≥10% weight increase one year after transplantation (Table 5) was 6 (obtained for sample B, SNP group#1). In sample A, the NNG was 13 for SNP group#2 and 24 for SNP group#1.

**Table 5:** Comparison of genetic versus non-genetic model for 10% weight gain prediction at one year after transplantation.

		AUROC [95% CI]	Specificity	Sensitivity	Accuracy	LRT-p	IDI [95% CI]*	NNG
**Sample A**	**SNP group#1**							
	*non genetic model*	0.66 [0.58–0.72]	0.59	0.66	0.61	0.0004	0.08 [0.06–0.10]	24
	*genetic model*	0.74 [0.70–0.83]	0.61	0.77	0.65			
	**SNP group#2**							
	*non genetic model*	0.66 [0.54–0.69]	0.65	0.62	0.64	0.008	0.17 [0.14–0.20]	13
	*genetic model*	0.80 [0.71–0.84]	0.70	0.77	0.72			
**Sample B**	**SNP group#1**							
	*non genetic model*	0.67 [0.61–0.88]	0.55	0.76	0.63	0.04	0.36 [0.28–0.45]	6
	*genetic model*	0.89 [0.79–0.97]	0.78	0.88	0.81			

AUROC: Area Under the Receiver Operating Characteristic Curve

IDI: Integrated Discrimination Improvement

NNG: Number Needed to Genotype

LRT: Likelihood Ratio tests

SNP: Single Nucleotide Polymorphism

CI: Confidence Interval

*p-value < 0.01

## Discussion

To our knowledge, this is the first study examining the association of clinical and genetic risk scores with WG in SOT patients. Our results showed that, in transplanted populations, previously GWAS-BMI related SNPs in general populations, were associated with BMI when combined in w-GRS. These results could be partly replicated in a second sample (i.e. sample B).

The influence of weighted score including SNP group#1 on BMI has been extensively replicated in several general populations from different ethnicities [29–32]. This is the first study evaluating the effect of these polymorphisms on BMI in SOT recipients (kidney, liver, lung, heart, or multi-organ) and WG, with positive results being found in both samples. SNPs group#2 was recently published [27] and contained a higher number of SNPs (including those from SNP group# 1 except of 2 SNPs). However, significant results were found only in sample A. The non replication using SNPs group#2 in sample B could be attributed either to no effect at all or to the low number of patients in the latter sample and the large number of polymorphisms in group#2, each one of small effect size, thus necessitating large sample sizes in order to observe an effect [48].

In addition, an exploratory analysis of 19 polymorphisms combined in a w-GRS (SNP group#3) showed an association with BMI in sample B. These variants were selected from a microarray study examining subcutaneous gene expression which was correlated with weight change in kidney transplant recipients [25]. These findings should be considered as preliminary as they were not further replicated nor corrected for multiple test. In sample B individuals were younger, had lower percentage of living donors and gained more weight after the first year of transplant compared to sample A. Young age, low BMI at baseline and deceased donors increase the risk of gaining weight, as previously described in the literature [15, 49]. Adding SNP group#3 to SNP group#1 resulted in an increased explained BMI variability of 4.1%. However, when all SNPs were combined (i.e. SNP group#2 and SNP group#3), no significant results were found, probably due to the low effect and sample size.

In a second step, we showed that a combination of extensive genetic factors and clinical data predicts better a 10% WG after the first year of treatment than considering the model with clinical data alone, increasing AUROC and accuracy. When examining genetic factors in sample A, several polymorphisms were significantly associated with 10% WG one year post-transplant. Interestingly, when looking at SNPs individually, only *MC4R (rs571312*, *rs6567160)* and *SEC16B (rs543874)* remained significant in both SNP group#1 and #2 analyses. *MC4R* is one of the most common genetic causes of obesity and this gene participates in appetite regulation and energy balance [50]. *SEC16B* has been associated with obesity-related phenotypes but the mechanism behind remains unknown. In sample B, 4 SNPs in or near *MTIF3*, *ETV5*, *GNPDA2* and *FAIM2* gene regions *(rs1006353*, *rs7647305*, *rs10938397* and *rs7138803)* were associated with 10% WG one year post-transplant (S8–S10 Tables). Most of these gene functions are not clear yet. *ETV5* modulates circulating glucocorticoids levels [51] and *GNPDA2* regulates metabolic pathways leading to insulin resistance [52]. Interestingly, the best group of polymorphisms predicting 10% WG at 12 months post-transplant was SNP group#2 (n = 97 SNPs) for sample A and SNP group#1 (n = 32 SNPs) for sample B. This could be tentatively explained by the fact that a higher sample size (i.e. sample A) is necessary to demonstrate the association with larger set of SNPs (i.e. SNP group#2). Finally, only the SNP group#1 was associated with BMI change over time in both samples A and B.

In samples A and B, the mean of WG after one year post-transplant is 3.5% and 6.3%, respectively, i.e. much lower than the 10% mean value described in the literature [15]. It should be noted that a solid consensus does not exist yet regarding WG after the first year post-transplantation; a mean of 10% has been described but a range from 3.5 to 10 kg as well. A WG of 10 kg over the first year following kidney [12, 13, 53] liver [14] and cardiac [10] transplantation as described in some studies would correspond to an increase of 14% of weight in our samples (considering a mean baseline weight in sample A and B of 71 kg and 69.5 kg, respectively) which would be much higher than the WG mean in our samples.

Some limitations of the present study should be acknowledged. These results can only be extrapolated to Caucasians. We could not obtain all genotypes, in particular those from the SNP group#3 and possible co-medications influencing weight in addition to the immunosuppressant treatment were not reported and/or considered. Finally, sample B size was small and other replication in larger cohorts should be tested. However, both samples were obtained from naturalistic setting studies, which should represent the real cases in clinical practice. Further studies should analyze whether graft rejection in less than one year would influence weight gain (out of the scope of the present study). Also, further analysis stratified by type of transplanted organ should be conducted, as weight gain may differ depending on this factor as recently described[54].

## Conclusions

To conclude, this is the first study evaluating extensively the association of w-GRS with BMI and the influence of clinical and genetic components on *≥*10% WG over the first year post transplant. The results obtained in the present study, showed the importance of integrating genetic factors in the final model, since they contain predictive information on *≥*10% WG. Genetics of obesity among SOT recipients remains an important issue and will definitely contribute towards treatment personalizing and prediction improvement of WG in these populations by identifying at risk-individuals.

## Supporting Information

S1 Fig Distribution of w-GRS within Samples A and B using SNP group#1 and #2. Upper: Sample A; Lower: Sample B.
(DOCX)

S1 Table SNP group#1 description. (DOCX)

S2 Table SNP group#2 description. (DOCX)

S3 Table SNP group#3 description. (DOCX)

S4 Table Distribution of glucocorticoid prescription (tglu) in individuals with less than 3 immunosuppressants. (DOCX)

S5 Table Distribution of glucocorticoid prescription (tglu) in individuals with 3 or more immunosuppressants. (DOCX)

S6 Table Weighted Genetic Risk Scores from candidate gene SNPs (SNP group#3) and their associations with BMI. (DOCX)

S7 Table Weighted genetic risk scores association with BMI in Sample B when combining GWAS with candidate gene SNPs. (DOCX)

S8 Table Estimates of the association analysis of 10% weight gain and individual SNP from group#1 in Sample A. (DOCX)

S9 Table Estimates of the association analysis of 10% weight gain and individual SNP from group#2 in Sample A. (DOCX)

S10 Table Estimates of the association analysis of 10% weight gain and individual SNP from group#1 in Sample B. (DOCX)
