# Supplementary material for: Weighted Genetic Risk Scores and Prediction of Weight Gain in Solid Organ Transplant Populations
Source: PLoS One. 2016 Oct 27;11(10):e0164443. doi: 10.1371/journal.pone.0164443 (PMC5082801; doi:10.1371/journal.pone.0164443)
Supplement: S1 Fig — Distribution of w-GRS within Samples A and B using SNP group#1 and #2. Upper: Sample A; Lower: Sample B. (DOCX) [file pone.0164443.s001.docx]

S1 Fig. Distribution of w-GRS within Samples A and B using SNP group#1 and #2


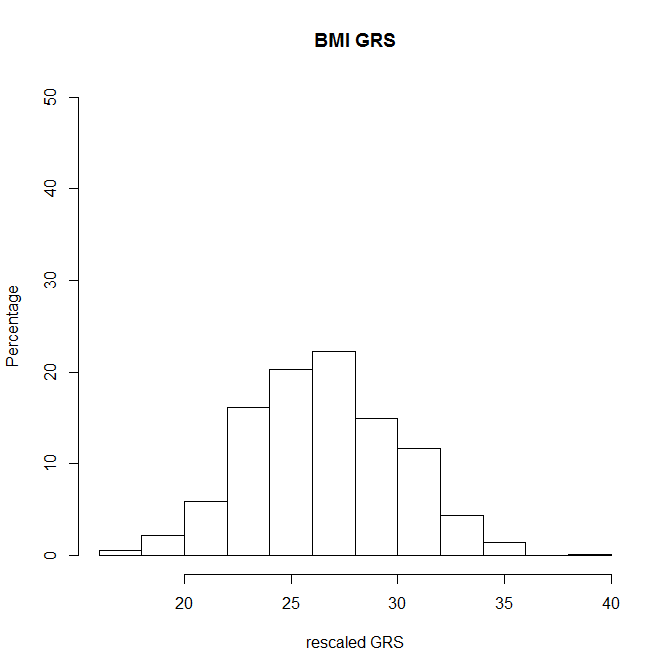


SNP group #1


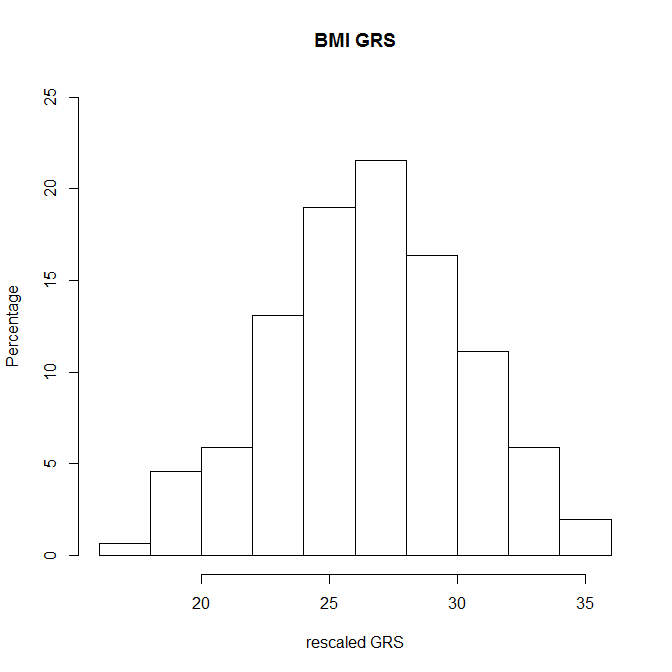


SNP group #1


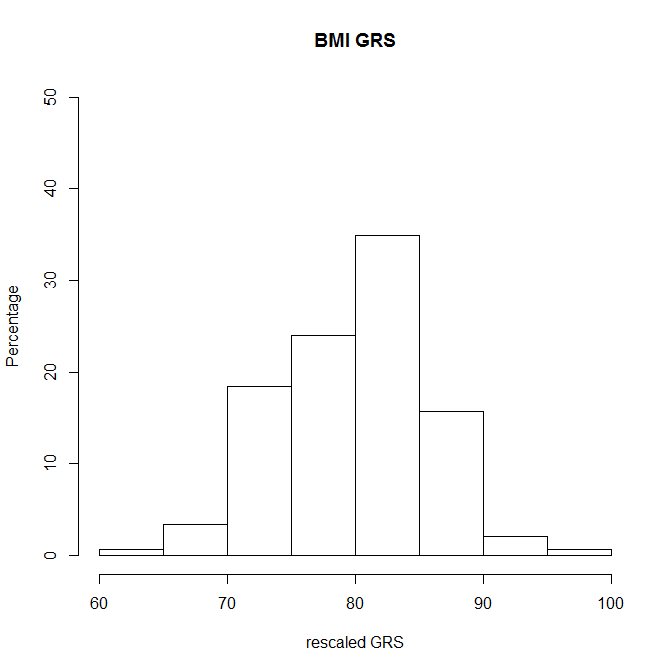


SNP group #2


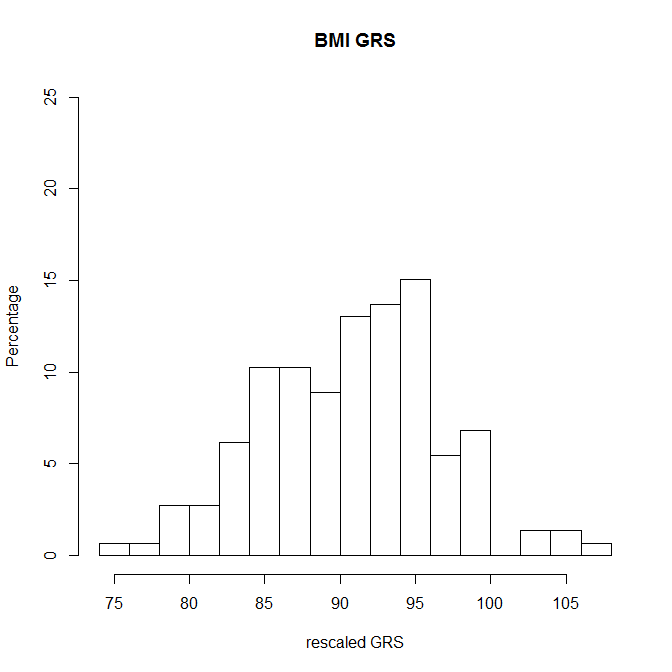


SNP group #2

*Upper: Sample A; Lower: Sample B*
