# Supplementary material for: Weighted Genetic Risk Scores and Prediction of Weight Gain in Solid Organ Transplant Populations
Source: PLoS One. 2016 Oct 27;11(10):e0164443. doi: 10.1371/journal.pone.0164443 (PMC5082801; doi:10.1371/journal.pone.0164443)
Supplement: S2 Table — (DOCX) [file pone.0164443.s003.docx]

S2 Table. SNP group#2 description (2)

| **SNP** | **Gene(s)** | **Proxy Sample A**  **(LD, r^2^)** | **Proxy Sample B (LD, r^2^)** | **Alleles** | **Effect allele** | **β** | **SE** | ***P* value** |
| --- | --- | --- | --- | --- | --- | --- | --- | --- |
| rs17024393 | *GNAT2*; *AMPD2* |  |  | C/T | T | 0.066 | 0.009 | 7.03E-14 |
| rs11847697 | *PRKD1* | not found in Sample A | rs10134820 (0.74) | T/C | C | 0.049 | 0.008 | 3.99E-09 |
| rs7899106 | *GRID1* | rs11201714 (1) |  | G/A | A | 0.04 | 0.007 | 2.96 × 10^−8^ |
| rs16851483 | *RASA2* |  | rs2035935 (0.91) | T/G | G | 0.048 | 0.008 | 3.55 × 10^−10^ |
| rs13107325 | *SLC39A8* |  |  | T/C | C | 0.048 | 0.007 | 1.83E-12 |
| rs11191560 | *NT5C2; CYP17A1*; *SFXN2* |  |  | C/T | T | 0.031 | 0.005 | 8.45 × 10^−9^ |
| rs12429545 | *OLFM4* |  |  | A/G | G | 0.033 | 0.005 | 1.09E-12 |
| rs13201877 | *IFNGR1*; *OLIG3* | not found in Sample A |  | G/A | A | 0.024 | 0.004 | 4.29 × 10^−8^ |
| rs2121279 | *LRP1B* |  |  | T/C | C | 0.025 | 0.004 | 2.31E-08 |
| rs17001654 | *NUP54*; *SCARB2* | rs17001561 (1) | rs17001561 (1) | G/C | C | 0.031 | 0.005 | 7.76 × 10^−9^ |
| rs2207139 | *TFAP2B* | rs943005 (1) | rs734597 (0.90) | G/A | A | 0.045 | 0.004 | 4.13E-29 |
| rs1460676 | *FIGN* | rs10192119 (1) |  | C/T | T | 0.021 | 0.004 | 4.98 × 10^−8^ |
| rs2245368 | *PMS2L11* | not found in Sample A | bad genotype quality | C/T | T | 0.032 | 0.006 | 3.19 × 10^−8^ |
| rs543874 | *SEC16B* |  |  | G/A | A | 0.048 | 0.004 | 2.62E-35 |
| rs17203016 | *CREB1*; *KLF7* |  |  | G/A | A | 0.021 | 0.004 | 3.41 × 10^−8^ |
| rs13078960 | *CADM2* | rs9852127 (0.95) | rs7622475 (0.95) | G/T | T | 0.03 | 0.004 | 1.74E-14 |
| rs12016871 | *MTIF3*; *GTF3A* |  | rs1885988 (0.82) | T/C | C | 0.03 | 0.005 | 2.29E-10 |
| rs17094222 | *HIF1AN* | rs17113301 (0.90) |  | C/T | T | 0.025 | 0.004 | 5.94 × 10^−11^ |
| rs9914578 | *SMG6*; *N29617* | rs8082647 (1) |  | G/C | C | 0.02 | 0.004 | 2.07 × 10^−8^ |
| rs6567160 | *MC4R* |  |  | C/T | T | 0.056 | 0.004 | 3.93E-53 |
| rs2176598 | *HSD17B12* |  |  | T/C | C | 0.02 | 0.004 | 2.97 × 10^−8^ |
| rs758747 | *NLRC3* |  |  | T/C | C | 0.023 | 0.004 | 7.47 × 10^−10^ |
| rs205262 | *C6orf106*; *SNRPC* |  |  | G/A | A | 0.022 | 0.004 | 1.75E-10 |
| rs11126666 | *KCNK3* |  |  | A/G | G | 0.021 | 0.003 | 1.33 × 10^−9^ |
| rs1016287 | *LINC01122* |  |  | T/C | C | 0.023 | 0.003 | 2.25E-11 |
| rs2033529 | *TDRG1; LRFN2* |  |  | G/A | A | 0.019 | 0.003 | 1.39 × 10^−8^ |
| rs2650492 | *SBK1*; *APOBR* | not found in Sample A |  | A/G | G | 0.021 | 0.004 | 1.92 × 10^−9^ |
| rs6465468 | *ASB4* | rs2375019 (0.80) |  | T/G | G | 0.025 | 0.005 | 4.98 × 10^−8^ |
| rs10968576 | *LINGO2* |  |  | G/A | A | 0.025 | 0.003 | 6.61E-14 |
| rs1000940 | *RABEP1* | rs3026101 (1) |  | G/A | A | 0.019 | 0.003 | 1.28 × 10^−8^ |
| rs12401738 | *FUBP1*; *USP33* | rs17381664 (0.83) |  | A/G | G | 0.021 | 0.003 | 1.15E-10 |
| rs3849570 | *GBE1* | rs3772883 (1) | rs6792696 (0.70) | A/C | C | 0.019 | 0.003 | 2.60 × 10^−8^ |
| rs6477694 | *EPB41L4B; C9orf4* | not found in Sample A |  | C/T | T | 0.017 | 0.003 | 2.67 × 10^−8^ |
| rs2176040 | *LOC646736*; *IRS1* | rs2943641 (0.96) | rs2972143 (0.96) | A/G | G | 0.024 | 0.004 | 9.99 × 10^−9^ |
| rs7138803 | *BCDIN3D*; *FAIM2* |  |  | A/G | G | 0.032 | 0.003 | 8.15E-24 |
| rs7239883 | *LOC284260; RIT2* |  |  | G/A | A | 0.023 | 0.004 | 1.51 × 10^−8^ |
| rs657452 | *AGBL4* |  |  | A/G | G | 0.023 | 0.003 | 5.48 × 10^−13^ |
| rs11583200 | *ELAVL4* |  |  | C/T | T | 0.018 | 0.003 | 1.48 × 10^−8^ |
| rs3888190 | *ATXN2L*; *SBK1*; *SULT1A2*; TUFM |  |  | A/C | C | 0.031 | 0.003 | 3.14E-23 |
| rs977747 | *TAL1* |  |  | T/G | G | 0.017 | 0.003 | 2.18 × 10^−8^ |
| rs3817334 | *MTCH2*; *C1QTNF4*; *SPI1*; CELF1 | rs7124681 (1) |  | T/C | C | 0.026 | 0.003 | 5.15E-17 |
| rs2080454 | *CBLN1* |  |  | C/A | A | 0.017 | 0.003 | 8.60 × 10^−9^ |
| rs1558902 | *FTO* | rs1421085 (1) | rs1421085 (1) | A/T | T | 0.082 | 0.003 | 7.51E-153 |
| rs7715256 | *GALNT10* | rs7719067 (1) |  | G/T | T | 0.017 | 0.003 | 8.85 × 10^−9^ |
| rs492400 | *PLCD4*; *CYP27A1*; USP37; *TTLL4*;*STK36*; *ZNF142*; *RQCD1* |  |  | C/T | T | 0.024 | 0.004 | 6.78 × 10^−9^ |
| rs9641123 | *CALCR*; *hsa-miR-653* | rs10488551 (0.93) | rs5014937 (0.70) | C/G | G | 0.029 | 0.005 | 2.08 × 10^−10^ |
| rs10938397 | *GNPDA2*; *GABRG1* |  |  | G/A | A | 0.04 | 0.003 | 3.21E-38 |
| rs12566985 | *FPGT-TNNI3K* | rs6604872 (1) | rs1514175 (0.97) | G/A | A | 0.024 | 0.003 | 3.28E-15 |
| rs9540493 | *MIR548X2*; *PCDH9* |  |  | A/G | G | 0.021 | 0.004 | 4.97 × 10^−8^ |
| rs3736485 | *SCG3*; *DMXL2* |  |  | A/G | G | 0.018 | 0.003 | 7.41 × 10^−9^ |
| rs10182181 | *ADCY3*; *POMC*; NCOA1;SH2B1; APOBR | rs713587 (1) |  | G/A | A | 0.031 | 0.003 | 8.78E-24 |
| rs10733682 | *LMX1B* |  |  | A/G | G | 0.017 | 0.003 | 1.83 × 10^−8^ |
| rs4787491 | *MAPK3*; *KCTD13*; *INO80E*; *TAOK2*; *YPEL3*; *DOC2A*; *FAM57B* |  |  | G/A | G | 0.022 | 0.004 | 2.70 × 10^−8^ |
| rs12286929 | *CADM1* |  |  | G/A | G | 0.022 | 0.003 | 1.31 × 10^−12^ |
| rs11688816 | *EHBP1* | rs360791 (0.90) |  | G/A | G | 0.017 | 0.003 | 1.89 × 10^−8^ |
| rs7141420 | *NRXN3* |  |  | T/C | T | 0.024 | 0.003 | 1.23E-14 |
| rs1808579 | *NPC1*; *C18orf8* | rs11663558 (1) |  | C/T | T | 0.017 | 0.003 | 4.17E-08 |
| rs4740619 | *C9orf93* |  |  | T/C | C | 0.018 | 0.003 | 4.56 × 10^−9^ |
| rs1928295 | *TLR4* |  |  | T/C | C | 0.019 | 0.003 | 7.91 × 10^−10^ |
| rs1167827 | *HIP1*; *PMS2L3*; *PMS2P5*; *WBSCR16* |  |  | G/A | A | 0.02 | 0.003 | 6.33 × 10^−10^ |
| rs2820292 | *NAV1* | rs1032524 (1) |  | C/A | A | 0.02 | 0.003 | 1.83 × 10^−10^ |
| rs12940622 | *RPTOR* |  |  | G/A | A | 0.018 | 0.003 | 2.49E-09 |
| rs6804842 | *RARB* |  |  | G/A | A | 0.019 | 0.003 | 2.48 × 10^−9^ |
| rs2365389 | *FHIT* | rs815710 (0.96) |  | C/T | T | 0.02 | 0.003 | 1.63 × 10^−10^ |
| rs11165643 | *PTBP2* | rs10489741 (1) |  | T/C | C | 0.022 | 0.003 | 2.07E-12 |
| rs2836754 | *ETS2* |  |  | C/T | T | 0.017 | 0.003 | 1.61 × 10^−8^ |
| rs3101336 | N*EGR1* |  |  | C/T | T | 0.033 | 0.003 | 2.66E-26 |
| rs1441264 | *MIR548A2* |  |  | A/G | G | 0.017 | 0.003 | 2.96 × 10^−8^ |
| rs9925964 | *KAT8;ZNF646*; *VKORC1*; *ZNF668*; *STX1B*;*FBXL19* | rs1978487 (0.98) |  | A/G | G | 0.019 | 0.003 | 8.11 × 10^−10^ |
| rs2112347 | *POC5*; *HMGCR*; *COL4A3BP* | rs10057967 (1) |  | T/G | G | 0.026 | 0.003 | 6.19E-17 |
| rs1528435 | *UBE2E3* | rs6727573 (1) |  | T/C | C | 0.018 | 0.003 | 1.20 × 10^−8^ |
| rs12885454 | *PRKD1* | rs11625899 (1) |  | C/A | A | 0.021 | 0.003 | 1.94 × 10^−10^ |
| rs4256980 | *TRIM66*; *TUB* | rs4929927 (1) | rs7113874 (1) | G/C | C | 0.021 | 0.003 | 2.90E-11 |
| rs3810291 | *ZC3H4* |  |  | A/G | G | 0.028 | 0.004 | 4.81E-15 |
| rs29941 | *KCTD15* | rs29942 (1) |  | G/A | A | 0.018 | 0.003 | 2.41E-08 |
| rs7164727 | *LOC100287559*; *BBS4* | rs9460 (0.84) |  | T/C | C | 0.019 | 0.003 | 3.92 × 10^−9^ |
| rs10132280 | *STXBP6* |  |  | C/A | A | 0.023 | 0.003 | 1.14 × 10^−11^ |
| rs9400239 | *FOXO3*; *HSS00296402* | not found in Sample A |  | C/T | T | 0.019 | 0.003 | 1.61 × 10^−8^ |
| rs17405819 | *HNF4G* | rs12679314 (1) |  | T/C | C | 0.022 | 0.003 | 2.07E-11 |
| rs7903146 | *TCF7L2* |  |  | C/T | T | 0.023 | 0.003 | 1.11 × 10^−11^ |
| rs6091540 | *ZFP64* | rs6096969 (1) |  | C/T | T | 0.03 | 0.004 | 2.15 × 10^−11^ |
| rs7599312 | *ERBB4* |  |  | G/A | A | 0.022 | 0.003 | 1.17 × 10^−10^ |
| rs9374842 | *LOC285762;* | rs1329530 (1) |  | T/C | C | 0.023 | 0.004 | 2.67 × 10^−8^ |
| rs17724992 | *GDF15*; *PGPEP1* |  |  | A/G | G | 0.019 | 0.004 | 3.42 × 10^−8^ |
| rs2033732 | *RALYL* |  |  | C/T | T | 0.019 | 0.004 | 4.89 × 10^−8^ |
| rs16951275 | *M4P2K5*; *LBXCOR1* | rs4776982 (1) | rs2241420 (1) | T/C | C | 0.031 | 0.004 | 1.91E-17 |
| rs11030104 | *BDNF* |  | rs7103411 (0.91) | A/G | G | 0.041 | 0.004 | 5.56E-28 |
| rs2287019 | *QPCTL*; *GIPR* |  |  | C/T | T | 0.036 | 0.004 | 4.59E-18 |
| rs7243357 | *GRP* |  |  | T/G | G | 0.022 | 0.004 | 3.86 × 10^−8^ |
| rs13021737 | *TMEM18* | not found in Sample A |  | G/A | A | 0.06 | 0.004 | 1.11E-50 |
| rs2075650 | *TOMM40*; *APOE*; *APOC1* |  |  | A/G | G | 0.026 | 0.005 | 1.25E-08 |
| rs12446632 | *GPRC5B*; *IQCK* | rs12444979 (0.88) |  | G/A | A | 0.04 | 0.005 | 1.48E-18 |
| rs1516725 | *E7V5* | rs10513801 (1) | rs4234589 (1) | C/T | T | 0.045 | 0.005 | 1.89E-22 |
| rs13191362 | *PARK2* | rs13202339 (1) |  | A/G | G | 0.028 | 0.005 | 7.34 × 10^−9^ |
| rs11057405 | *CLIP1* |  |  | G/A | A | 0.031 | 0.006 | 2.02 × 10^−8^ |
| rs11727676 | *HHIP* |  |  | T/C | C | 0.036 | 0.006 | 2.55 × 10^−8^ |
| rs16907751 | *ZBTB10* | not found in Sample A |  | C/T | T | 0.047 | 0.009 | 3.89 × 10^−8^ |

*SE: Standard Error*

(2) Locke AE, Kahali B, Berndt SI, Justice AE, Pers TH, Day FR, et al. Genetic studies of body mass index yield new insights for obesity biology. Nature. 2015;518(7538):197-206
