# Supplementary material for: Weighted Genetic Risk Scores and Prediction of Weight Gain in Solid Organ Transplant Populations
Source: PLoS One. 2016 Oct 27;11(10):e0164443. doi: 10.1371/journal.pone.0164443 (PMC5082801; doi:10.1371/journal.pone.0164443)
Supplement: S3 Table — (DOCX) [file pone.0164443.s004.docx]

S3 Table. SNP group#3 description (3)

| **Gene** | **SNP** | **Position** | **Major/minor allele** | **Effect allele** | **β-coefficients *** | **Proxy Sample A (LD, r^2^)** | **Proxy Sample B (LD, r^2^)** | **# SNPs tagged** |
| --- | --- | --- | --- | --- | --- | --- | --- | --- |
| MSRA | rs2001338 | intron-variant(dbSNP) | A/G | A | -0.0108 |  | rs13254942 (0.89) | 9 |
| NMUR2 | rs982716 | utr-variant-3-prime(dbSNP) | C/T | T | -0.0033 |  | rs17113291 (0.82) | 5 |
| FSD2 | rs12592976 | intron-variant(dbSNP) | T/C | T | -0.0046 |  | rs17158366 (0.80) | 1 |
| REPIN1 | rs1051760 | utr-variant-3-prime(dbSNP) | A/G | A | 0.0119 |  | rs17173681 (1) | 2 |
| ANGPTL2 | rs999092 | intron-variant(dbSNP) | A/G | A | 0.0096 | rs11789486 (1) | rs2789507 (0.78) | 5 |
| LEP | rs4236625 | intron-variant(dbSNP) | A/T | A | -0.0133 | rs7795794 (0.88) | rs4731427 (0.80) | 3 |
| GLIS3 | rs7870193 | intron-variant(dbSNP) | C/T | T | 0.0016 | rs2791757 (0.86) | rs605571 (1) | 4 |
| GRB14 | rs13000232 | intron-variant(dbSNP) | G/C | C | 0.0022 | rs4130269 (0.95) | rs6754749 (0.90) | 7 |
| TAS2R38 | rs1726866 | missense(GVS) | A/G | A | 0.0013 |  | rs713598 (0.73) | 2 |
| PTRF | rs12948909 | intron-variant(dbSNP) | A/C | A | -0.005 | rs7223784 (1) | rs7223784 (1) | 1 |
| BCMO1 | rs11865869 | intron-variant(dbSNP) | A/G | A | -0.0032 |  |  | 1 |
| CRP | rs1205 | utr-variant-3-prime(dbSNP) | C/T | T | 0.0077 |  |  | 0 |
| CPE | rs1438114 | intron-variant(dbSNP) | T/G | T | -0.0048 |  |  | 3 |
| EXT2 | rs2067787 | intron-variant(dbSNP) | T/C | T | -0.0046 |  |  | 0 |
| MTCH2 | rs3817334 | intron-variant(dbSNP) | T/C | C | 0.026 | rs7124681 (1) |  | 2 |
| SERPINA12 | rs4905211 | intron-variant(dbSNP) | G/A | A | -0.0027 |  |  | 0 |
| H6PD | rs732950 | intron-variant(dbSNP) | G/T | T | -0.0037 | rs2268175 (0.93) |  | 2 |
| TFAP2B | rs987237 | intron-variant(dbSNP) | A/G | G | 0.045 |  |  | 0 |
| HRASLS2 | rs9943597 | intron-variant(dbSNP) | A/C | A | 0.0032 |  |  | 2 |

** β-coefficients are obtained from GIANT consortia*

(3) Cashion A, Stanfill A, Thomas F, Xu L, Sutter T, Eason J, et al. Expression levels of obesity-related genes are associated with weight change in kidney transplant recipients. PloS one. 2013;8(3):e59962
