# Supplementary material for: Weighted Genetic Risk Scores and Prediction of Weight Gain in Solid Organ Transplant Populations
Source: PLoS One. 2016 Oct 27;11(10):e0164443. doi: 10.1371/journal.pone.0164443 (PMC5082801; doi:10.1371/journal.pone.0164443)
Supplement: S4 Table — (DOCX) [file pone.0164443.s005.docx]

S4 Table. Distribution of glucocorticoid prescription (tglu) in individuals with less than 3 immunosuppressants

| group

tglu | 1 2 | Total

-----------+----------------------+----------

0 | 296 68 | 364

| 72.55 54.84 | 68.42

-----------+----------------------+----------

1 | 112 56 | 168

| 27.45 45.16 | 31.58

-----------+----------------------+----------

Total | 408 124 | 532

| 100.00 100.00 | 100.00

Pearson chi2(1) = 13.8050 Pr = 0.000

group1: sample A, group2: sample B
