# Supplementary material for: Weighted Genetic Risk Scores and Prediction of Weight Gain in Solid Organ Transplant Populations
Source: PLoS One. 2016 Oct 27;11(10):e0164443. doi: 10.1371/journal.pone.0164443 (PMC5082801; doi:10.1371/journal.pone.0164443)
Supplement: S5 Table — (DOCX) [file pone.0164443.s006.docx]

S5 Table. Distribution of glucocorticoid prescription (tglu) in individuals with 3 or more immunosuppressants

| group

tglu | 1 2 | Total

-----------+----------------------+----------

0 | 2 0 | 2

| 0.36 0.00 | 0.32

-----------+----------------------+----------

1 | 551 81 | 632

| 99.64 100.00 | 99.68

-----------+----------------------+----------

Total | 553 81 | 634

| 100.00 100.00 | 100.00

Pearson chi2(1) = 0.2939 Pr = 0.588

group1: sample A, group2: sample B
