# Supplementary material for: Weighted Genetic Risk Scores and Prediction of Weight Gain in Solid Organ Transplant Populations
Source: PLoS One. 2016 Oct 27;11(10):e0164443. doi: 10.1371/journal.pone.0164443 (PMC5082801; doi:10.1371/journal.pone.0164443)
Supplement: S6 Table — (DOCX) [file pone.0164443.s007.docx]

S6 Table. Weighted Genetic Risk Scores from candidate gene SNPs (SNP group#3) and their associations with BMI.

|  | n | Effect on BMI per additional risk allele [CI 95%] | p-value* | E. Var (%) |
| --- | --- | --- | --- | --- |
| Sample A | 938 | 0.01 [-0.01 - 0.03] | 1.0 | n.c |
| Sample B | 118 | 0.05 [0.01 - 0.10] | **0.048** | 1.72 |

*E. Var: Explained Variability*

*CI: Confidence Interval*

*BMI: Body Mass Index*

*n.c: not calculated because of non significant association*

** multiple test correction p-value*
