# Supplementary material for: Weighted Genetic Risk Scores and Prediction of Weight Gain in Solid Organ Transplant Populations
Source: PLoS One. 2016 Oct 27;11(10):e0164443. doi: 10.1371/journal.pone.0164443 (PMC5082801; doi:10.1371/journal.pone.0164443)
Supplement: S7 Table — (DOCX) [file pone.0164443.s008.docx]

S7 Table. Weighted genetic risk scores association with BMI in Sample B when combining GWAS with candidate gene SNPs.

|  | n | effect on BMI per additional risk allele [CI 95%] | p-value | E. Var (%) |
| --- | --- | --- | --- | --- |
| ***SNP group#1 + SNP group#3*** | 115 | 0.16 [0.08 - 0.24] | 0.001 | 4.1 |
| ***SNP group#2 + SNP group#3*** | 108 | 0.04 [-0.04 - 0.11] | 0.11 | n.c |

*E. Var: Explained Variability*

*CI: Confidence Interval*

*BMI: Body Mass Index*

*SNP: Single Nucleotide Polymorphism*

*n.c: not calculated because of non significant association*
