# Supplementary material for: Weighted Genetic Risk Scores and Prediction of Weight Gain in Solid Organ Transplant Populations
Source: PLoS One. 2016 Oct 27;11(10):e0164443. doi: 10.1371/journal.pone.0164443 (PMC5082801; doi:10.1371/journal.pone.0164443)
Supplement: S8 Table — (DOCX) [file pone.0164443.s009.docx]

S8 Table. Estimates of the association analysis of 10% weight gain and individual SNP from group#1 (1) in Sample A.

| SNP | Estimate | p-value |
| --- | --- | --- |
| rs13078807 | -0.254795 | 0.16058 |
| rs1421085 | -0.094457 | 0.49556 |
| rs12444979 | -0.304122 | 0.16997 |
| rs7103411 | -0.375411 | **0.02578** |
| rs987237 | 0.015542 | 0.93038 |
| rs17109256 | -0.403029 | **0.02501** |
| rs571312 | 0.449263 | **0.00327** |
| rs2241423 | -0.199464 | 0.22487 |
| rs1514175 | -0.10445 | 0.4462 |
| rs543874 | 0.429708 | **0.01391** |
| rs13107325 | -0.540395 | 0.06087 |
| rs206936 | -0.3949 | **0.0173** |
| rs6864049 | 0.092884 | 0.5007 |
| rs9512699 | -0.084512 | 0.62025 |
| rs7124681 | 0.203121 | 0.12911 |
| rs10057967 | 0.013058 | 0.92649 |
| rs2867125 | -0.154048 | 0.3715 |
| rs3810291 | 0.010613 | 0.94625 |
| rs713587 | 0.160861 | 0.25175 |
| rs2815752 | 0.006356 | 0.96531 |
| rs29942 | -0.135384 | 0.35775 |
| rs10489741 | 0.188075 | 0.16046 |
| rs10938397 | 0.188506 | 0.14969 |
| rs11041994 | 0.208768 | 0.12103 |
| rs7138803 | -0.176378 | 0.22461 |
| rs1016287 | -0.275752 | 0.07385 |
| rs2287019 | -0.138718 | 0.43526 |
| rs10968576 | 0.150774 | 0.3055 |
| rs3888190 | -0.052741 | 0.71662 |

(1) Speliotes EK, Willer CJ, Berndt SI, Monda KL, Thorleifsson G, Jackson AU, et al. Association analyses of 249,796 individuals reveal 18 new loci associated with body mass index. Nat Genet. 2010;42(11):937-48
