# Supplementary material for: Weighted Genetic Risk Scores and Prediction of Weight Gain in Solid Organ Transplant Populations
Source: PLoS One. 2016 Oct 27;11(10):e0164443. doi: 10.1371/journal.pone.0164443 (PMC5082801; doi:10.1371/journal.pone.0164443)
Supplement: S9 Table — (DOCX) [file pone.0164443.s010.docx]

S9 Table. Estimates of the association analysis of 10% weight gain and individual SNP from group#2 (2) in Sample A.

| SNP | Estimate | p-value |
| --- | --- | --- |
| rs1421085 | -0.159025 | 0.307091 |
| rs10057967 | -0.094246 | 0.551969 |
| rs10192119 | -0.030546 | 0.880932 |
| rs1032524 | 0.24946 | 0.0961 |
| rs10488551 | 0.319874 | **0.034526** |
| rs10489741 | 0.074833 | 0.61701 |
| rs10513801 | 0.229087 | 0.321642 |
| rs11201714 | 0.031533 | 0.922691 |
| rs11625899 | -0.197364 | 0.251103 |
| rs11663558 | 0.207099 | 0.192148 |
| rs12444979 | -0.593364 | **0.017453** |
| rs12679314 | -0.022072 | 0.891643 |
| rs13202339 | -0.674293 | **0.009954** |
| rs1329530 | -0.238512 | 0.20058 |
| rs17001561 | 0.028612 | 0.889764 |
| rs17113301 | -0.285566 | 0.140003 |
| rs17381664 | 0.246713 | 0.128565 |
| rs1978487 | -0.069231 | 0.666198 |
| rs2375019 | -0.092106 | 0.553819 |
| rs2943641 | -0.185866 | 0.253314 |
| rs29942 | -0.267418 | 0.116416 |
| rs3026101 | -0.264773 | 0.107689 |
| rs360791 | -0.144297 | 0.345186 |
| rs3772883 | 0.18849 | 0.218581 |
| rs4776982 | -0.242288 | 0.190709 |
| rs4929927 | -0.22638 | 0.139041 |
| rs6096969 | -0.02305 | 0.891029 |
| rs6604872 | 0.046063 | 0.759113 |
| rs6727573 | -0.292718 | 0.059932 |
| rs7124681 | 0.433435 | **0.005844** |
| rs7719067 | 0.104034 | 0.493514 |
| rs8082647 | -0.178061 | 0.343131 |
| rs815710 | -0.308279 | **0.045592** |
| rs9460 | 0.042749 | 0.797482 |
| rs9852127 | -0.382151 | 0.063652 |
| rs6567160 | 0.57424 | **0.000982** |
| rs10938397 | 0.207515 | 0.154924 |
| rs543874 | 0.415116 | **0.036301** |
| rs943005 | 0.165221 | 0.438742 |
| rs11030104 | -0.363168 | 0.057979 |
| rs3101336 | 0.069145 | 0.674915 |
| rs7138803 | -0.207827 | 0.204084 |
| rs713587 | 0.201007 | 0.202867 |
| rs3888190 | -0.034906 | 0.828104 |
| rs2287019 | -0.139073 | 0.490072 |
| rs3810291 | 0.074855 | 0.676274 |
| rs7141420 | 0.159915 | 0.279451 |
| rs10968576 | 0.20887 | 0.217982 |
| rs17024393 | -0.440384 | 0.448754 |
| rs12429545 | -0.13429 | 0.582704 |
| rs13107325 | -0.616266 | 0.060426 |
| rs1016287 | -0.373499 | **0.02823** |
| rs205262 | 0.090695 | 0.584002 |
| rs12016871 | 0.03869 | 0.842219 |
| rs12940622 | 0.100696 | 0.505685 |
| rs2075650 | 0.148407 | 0.522673 |
| rs2121279 | 0.08535 | 0.706762 |
| rs657452 | 0.109197 | 0.622554 |
| rs12286929 | 0.004755 | 0.975411 |
| rs7903146 | 0.198284 | 0.179534 |
| rs10132280 | -0.142897 | 0.380187 |
| rs7599312 | 0.187752 | 0.273979 |
| rs16851483 | -0.118055 | 0.724854 |
| rs1167827 | -0.126642 | 0.405741 |
| rs758747 | -0.448849 | **0.008011** |
| rs1928295 | -0.102184 | 0.525349 |
| rs11126666 | 0.189335 | 0.299881 |
| rs6804842 | 0.221691 | 0.149383 |
| rs4740619 | -0.040525 | 0.79004 |
| rs3736485 | 0.074666 | 0.62513 |
| rs11191560 | -0.255952 | 0.306483 |
| rs2033529 | 0.139119 | 0.381807 |
| rs11583200 | 0.071722 | 0.743719 |
| rs10733682 | -0.269454 | 0.078397 |
| rs11057405 | 0.130634 | 0.634365 |
| rs11727676 | -0.109355 | 0.704489 |
| rs2176598 | -0.239284 | 0.194432 |
| rs17724992 | 0.133237 | 0.441072 |
| rs7243357 | -0.232786 | 0.227052 |
| rs2033732 | -0.298565 | 0.110411 |
| rs492400 | 0.006367 | 0.966948 |
| rs2080454 | -0.062709 | 0.702101 |
| rs7239883 | 0.028606 | 0.85221 |
| rs2836754 | 0.010743 | 0.945502 |
| rs977747 | 0.222224 | 0.159258 |
| rs4787491 | -0.258058 | 0.097914 |
| rs1441264 | 0.077195 | 0.616131 |
| rs17203016 | -0.276009 | 0.212814 |
| rs9540493 | -0.261961 | 0.096828 |

(2) Locke AE, Kahali B, Berndt SI, Justice AE, Pers TH, Day FR, et al. Genetic studies of body mass index yield new insights for obesity biology. Nature. 2015;518(7538):197-206
